# Pediatric Cardiology in the Age of Artificial Intelligence: An Updated Review of Bioethical Considerations and Impact on Care Quality

**DOI:** 10.7759/cureus.107313

**Published:** 2026-04-18

**Authors:** Maria-Guadalupe Jimenez-Carbajal, Ernesto Roldan-Valadez, Fabiola Lopez-Madrigal

**Affiliations:** 1 Department of Pediatric Interventional Cardiology, Unidad Cardiologica Infantil Mexico, Mexico City, MEX; 2 Division of Research, Instituto Nacional De Rehabilitación "Luis Guillermo Ibarra Ibarra" (INR), Mexico City, MEX; 3 Department of Radiology, I.M. Sechenov First Moscow State Medical University (Sechenov University), Moscow, RUS

**Keywords:** ai bioethics, algorithmic fairness, artificial intelligence, bioethics framework, clinical decision support systems (cdss), intervention pediatric cardiology, pediatric congenital heart disease, quality of healthcare

## Abstract

Innovations in technology continue to create new possibilities in all areas of medicine, including pediatric cardiology. Some of these possibilities include earlier diagnosis, clinical decision support, risk assessment, and image interpretation. As artificial intelligence (AI)-integrated systems transition from research to routine practice, they influence employee decision-making and raise concerns about patient safety, data privacy, clinician control, and accountability, especially in decisions impacting minors and their families.

This article is presented as a narrative, ethics- and quality-focused review rather than a formal systematic review or meta-analysis. We utilize the review framework provided by the World Health Organization (WHO) and the Institute of Medicine (IOM), focusing on the six domains of quality: safety, effectiveness, patient-centeredness, timeliness, efficiency, and equity. We address current applications of AI in pediatric cardiology, including echocardiography, electrocardiography, AI-based detection in congenital heart disease, and prognostic modeling in the ICU.

We address pediatric algorithmic bias, explainability, and consent, which are among the most challenging bioethical issues when framing an AI tool as appropriate for bedside use. Consistency across use cases is essential; AI should strengthen, not replace, clinical reasoning and responsibility.

## Introduction and background

Most fields of medicine are influenced and reshaped by rapid advancements in technology, and pediatric cardiology is no different. While the rate of development in artificial intelligence (AI) is extremely rapid, its implementation in healthcare represents a significant challenge, especially when it comes to integrating it into daily clinical practice, and leaves several ethical, legal, and equity-related questions unresolved. These concerns are particularly prominent in settings with little to no infrastructure and limited digital access.

**Figure 1:**
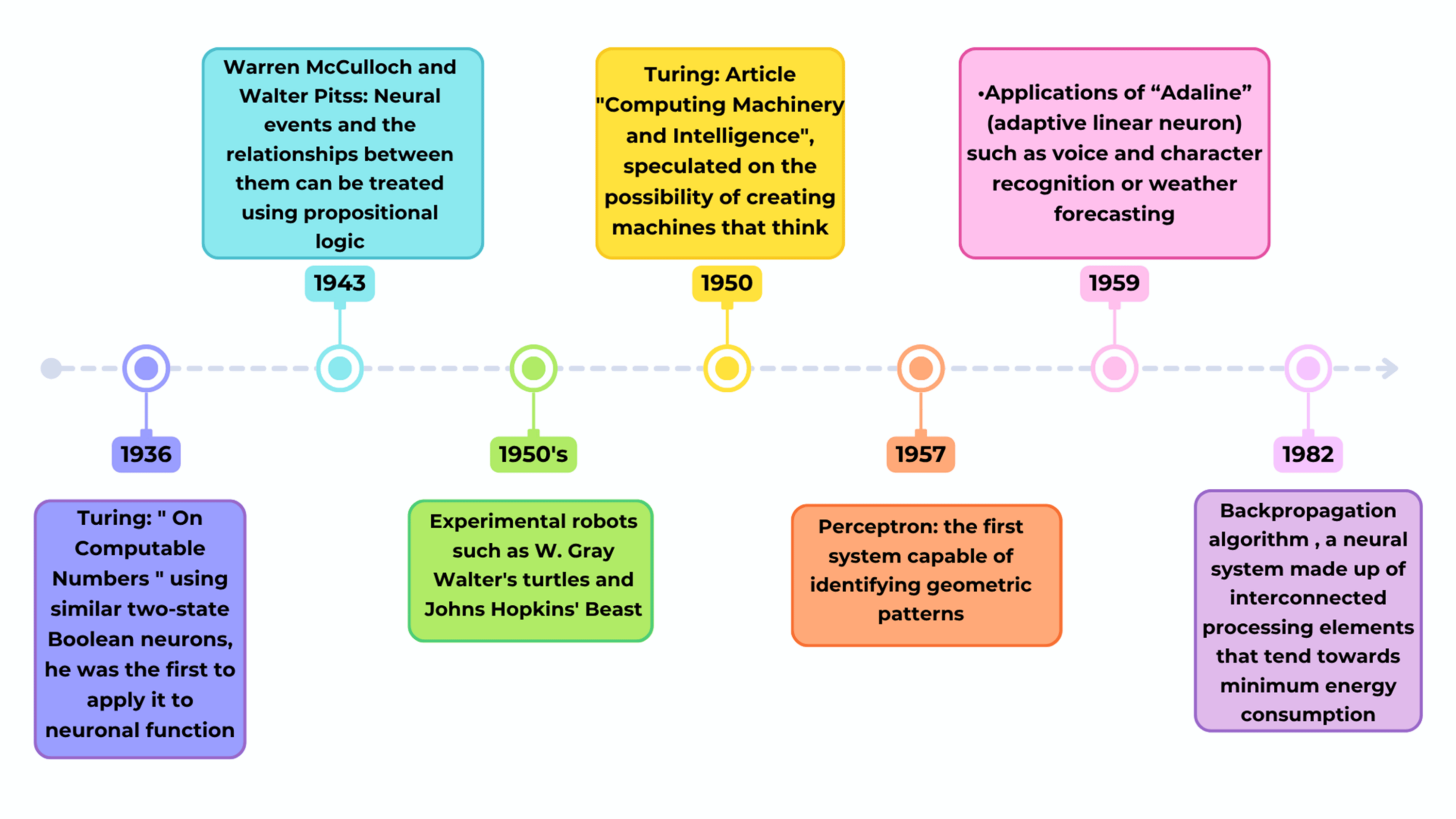
Evolution of foundational ideas in AI This timeline is based on the evolution of AI. It draws on the work of pioneers such as Alan Turing, Warren McCulloch, and Walter Pitts, as well as early robotic systems. Turing (1936) wrote an article on “computable numbers,” which is considered a milestone in AI history. Other accomplishments include the development of logical models (positive and negative) for neural functions and the perceptron neural model. These developments created the basis for later inventions in machine learning, including the ADALINE system and the backpropagation algorithm. These systems, in combination with others, are rapidly changing AI applications across many industries, including pediatric cardiology. Image credits: Created by María-Guadalupe Jiménez-Carbajal using Canva (Canva Pty Ltd., Sydney, Australia). AI: artificial intelligence; ADALINE: adaptive linear neuron

These aspects are highly relevant in pediatrics, an area in which clinicians are currently trying to integrate AI tools to support decision-making in fields as complex as pediatric cardiology, ranging from fetal-stage evaluations to infants and children with complex heart defects, as well as children with acquired heart disease. Although the systematic use of these tools may contribute to improvements in diagnostic accuracy and workflows, their use entails potential responsibilities in terms of patient safety within the populations in which they are applied (Figure 1).

This review is intended to analyze the AI technologies available in pediatric cardiology to determine whether AI can be used to improve the quality of care while remaining within the boundaries of the core principles of bioethics. It highlights the existing technologies in pediatric cardiology, particularly the bioethical principles and governance structures that apply, and the importance of collaboration between ethics, technology, and policy. Because care decisions involve families, the review also stresses clear, empathic communication and shared decision-making.

This narrative review was carried out through a specific bibliographic search of the PubMed and Google Scholar databases of medical literature, using combined terms such as "artificial intelligence," "pediatric cardiology," "congenital heart disease," "electrocardiography," "echocardiography," "magnetic resonance imaging," "tomography," "bioethics," and "quality of care," considering articles in English from 2020 to 2025, with the inclusion of literature from previous years based on its scientific, legal, and international normative importance. We did not perform a risk-of-bias analysis or a quantitative analysis.

Adopting ethical AI technologies for healthcare in pediatrics should prioritize governance that respects people, is open, accountable, and equitable, as recommended by the World Health Organization (WHO) AI guidelines [1]. Bridging both the digital and ethical gaps requires education, practical guidance, and policy interventions directed at structural inequities that disproportionately affect children with heart disease.

## Review

The expansion of artificial intelligence within the medical field

Core Concepts of Artificial Intelligence and Medicine

AI is a branch of computer science that involves designing systems that can learn and perform complex tasks autonomously, such as problem structuring, understanding, decision-making, or problem solving, even without the need for step-by-step human instructions [2,3].

Its principles are largely based on models of human thinking, as described by Mahoney, who proposed that humans can be seen as information processors who respond to stimuli, which leads to learning and behavioral changes, often through repetition, establishing patterns that will influence future decision-making [4]. Thought processes and mechanisms reside in the brain's neural networks, which have inspired many computational models, as shown in Figure 1 [5].

AI incorporates other systems and methodologies such as deep learning and data science. This includes large multimodal models that are able to combine and analyze various types of data, such as text, images, audio, video, and other sensory data, in order to assist in medical diagnosis, decision support, and personal care [3,6]. Another central area is machine learning, which is concerned with devising algorithms through which systems gain the ability to learn from experience and modify their conclusions without being programmed to do so [7].

AI system training can be conducted through supervised learning, in which a person guides the system by defining and organizing a dataset, allowing algorithms to be designed for decision-making, or through unsupervised learning, where the system can learn on its own by recognizing patterns in a dataset with only limited guidance and some training in algorithms [5].

Uses of Artificial Intelligence in Modern Medicine and Benefits 

In previous decades, the medical field has achieved a lot with the use of AI tools, which is especially evident in the areas of diagnosis and patient management. In the new paradigm of precision medicine, AI algorithms can analyze large amounts of data and identify diseases at earlier stages as a result of recognizing patterns, analyzing images, and providing support in real time for diagnosis and treatment decision-making. These tools also change the way X-rays and other imaging studies, including ultrasounds, CT scans, and MRIs, are analyzed.

AI tools such as chatbots and virtual assistants have also improved operational efficiency. These technologies aid in the optimization of workflows in hospitals and clinics by automating processes such as appointment scheduling, tracking bed occupancy, and verifying insurance information.

AI is being incorporated into teaching, assessment, and management of resources at medical schools. Integrated simulation, gamification, and smart educational systems create interactive, personalized learning. Significantly, the data generated from these systems is an important resource for research and development in clinical medicine [8].

Bioethical challenges of artificial intelligence in pediatric healthcare

With every new technological advancement, new ethical questions arise around the foundations of clinical decision-making, especially regarding AI in healthcare. Amor Villalpando defines ethics as “the practical and normative science that rationally studies the goodness and badness of human acts” [9]. Bioethics, as a discipline, emerged in the 20th century as the study of systematic human conduct in the life sciences and the dilemmas surrounding health and human dignity [9]. Its foundations must be reconsidered in response to the potential impact of technological evolution, including machine learning, deep learning, and artificial neural networks used in the analysis, interpretation, and manipulation of large and complex datasets.

The four basic principles of bioethics, autonomy, beneficence, non-maleficence, and justice, were first introduced by Beauchamp and Childress in their book Principles of Biomedical Ethics [10]. These principles are based on the Belmont Report, which outlines fundamental ethical aspects of human subjects research, including respect for persons, beneficence, and justice [11].

Autonomy within healthcare systems requires that practitioners furnish patients with all relevant information and that the information given is easy to understand and comprehend, to facilitate participation on the patient’s part and empower them to make voluntary choices and take part in decision-making.

Exercising beneficence and non-maleficence means constantly weighing the potential risks and benefits of any medical intervention in order to avoid harm and maximize patient benefit. Justice, specifically, distributive justice, requires that there be no barriers to receiving care and that no individual or group be discriminated against, exploited, or neglected [10,12].

The use of large sets of data and learning models can result in negative outcomes, including algorithmic discrimination, violation of privacy, and lack of transparency in how decisions are made, sometimes referred to as the “black box” problem of deep learning systems [13]. Such situations have led to the development of governance frameworks and models for the ethical use of AI, with a focus on issues such as transparency, accountability, oversight, and the right to equitable access [14].

In the 2021 Guidance on Ethics and Governance of AI for Health, the WHO states six ethical principles that should guide the implementation of AI healthcare systems [1]. These include protecting human autonomy through the appropriate use of technology to assist human decision-making and not to supplant it; advocating for the promotion of human health and the public good by supporting safety, quality, and risk control compliance; the defense of the right to understand and know about AI processes for both providers and patients; promotion of responsibility and accountability, encouraging the responsible use of information technologies; ensuring equitable inclusion, use, and access to AI technologies by promoting the active and joint participation of developers, promoters, and beneficiaries, including those belonging to vulnerable groups, seeking to avoid or minimize all types of algorithmic biases; and promotion of responsive and sustainable AI with continuous assessments of the changes and needs of the sectors where it is to be applied, while at the same time minimizing its environmental footprint.

When AI technologies and techniques are integrated into healthcare for children, ethics take on a new level of significance. This is, in part, due to the fact that children are not only developmentally less mature but also legally dependent. Decision-making is often a shared process, also involving parents or guardians. This is especially true for children in the healthcare system who are most vulnerable and underserved, and therefore, the potential for harm or inequity is further exacerbated.

In this way, inequitable access to the benefits of AI in healthcare, combined with child healthcare, holds significant ethical complexity.

The adoption of AI across medical specialties is fairly rapid, and pediatrics, in particular, is no exception. As per a bibliometric analysis by Galdo and colleagues, published in 2024, out of 19 medical specialties, pediatrics is ranked seventh in terms of scientific output in the field of AI [15]. The authors propose a framework called the “7Ps of Medicine”, which includes Predictive, Preventive, Personalized, Precise, Participatory, Peripheral, and Polyprofessional, as guiding principles for the responsible use of AI in pediatric medicine.

The pediatric population is perhaps one of the most varied, as it encompasses a broad range of developmental stages, from infancy through adolescence. In this age group, AI applications have targeted the assistive and supportive early stages of multi-system, age-group, and cross-organ disease diagnostics. In the past, diagnostic tools and nutritional assessment for therapeutic planning included anthropometric tables that measured weight, height, and body surface area. Now, even basic digital applications can assess a child’s nutrition and hydration status by inputting three simple variables, such as date of birth, weight, and height. Nowadays, there are applications that include tools for the detection of neonatal jaundice; early autism detection through AI-driven MRI analysis; tools that can predict the risk of death due to sepsis; and algorithms for estimating bone age [15].

**Figure 2:**
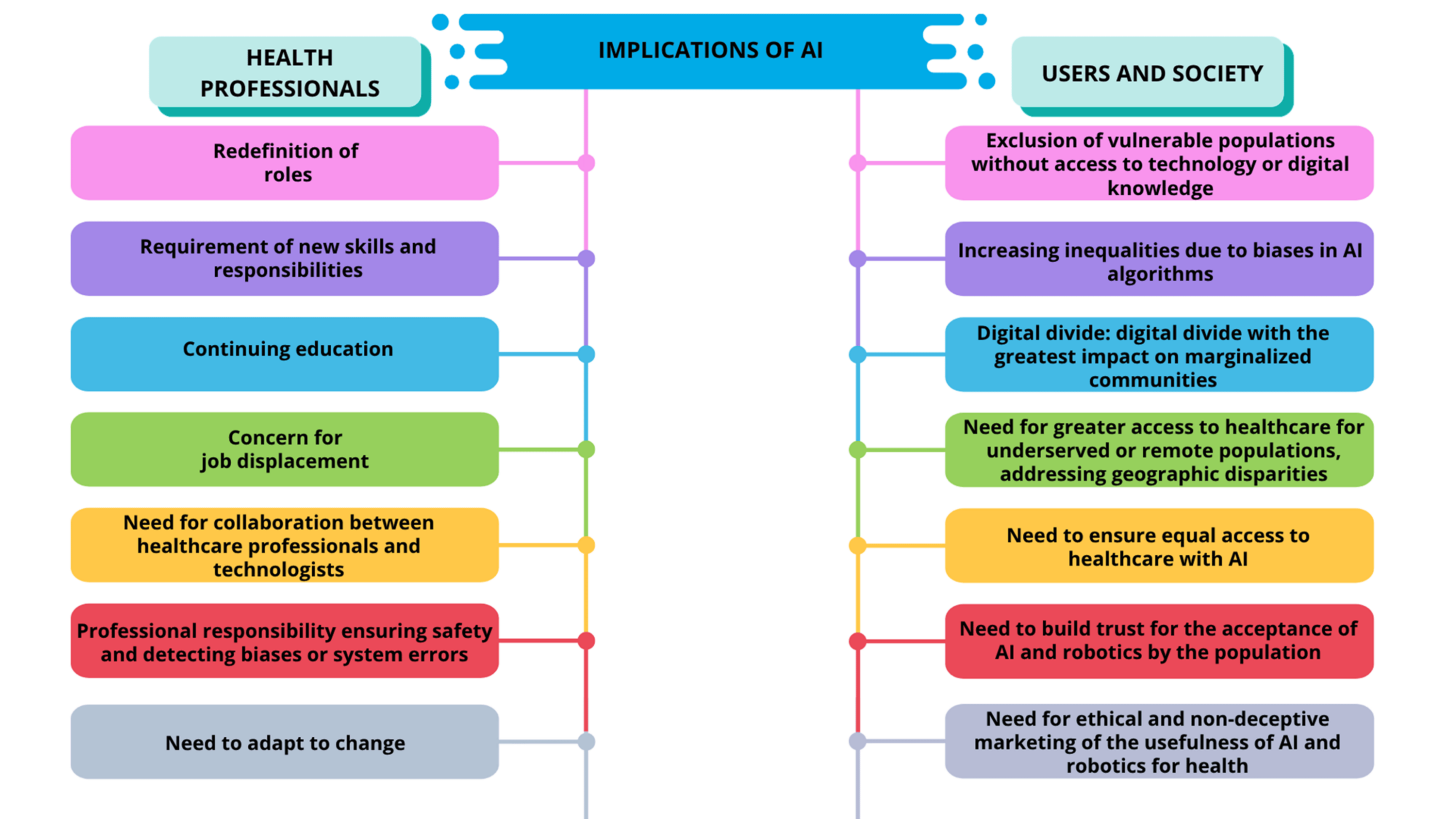
Ethical and professional implications of AI in healthcare: perspectives of health professionals and society This figure captures the social, ethical, and professional impacts of AI in medicine. Using AI in medicine may transform the roles of clinicians and redefine what is expected of them. It will create a need for new training and adult learning while raising the specter of job loss and the obsolescence of certain positions. There is also a need for different professional roles to work together, as well as for ethical considerations surrounding practitioner professionalism related to safety, bias, and system errors. The figure describes implications, which include the lack of inclusion of socially and digitally excluded individuals, the worsening of algorithmic discrimination, and digital inequity. It also suggests the importance of justice in AI healthcare, rights in digital communication, and public confidence in AI and robotics in a socially just manner. These issues reflect concerns of technological justice, as well as ethical concerns regarding technology in pediatric cardiology and medicine overall. Image credits: Created by María-Guadalupe Jiménez-Carbajal using Canva (Canva Pty Ltd., Sydney, Australia). AI: artificial intelligence

These innovations have the potential to be of clinical significance, as well as to serve as research tools for constructing decision-support systems for children, as shown in Figure 2.

The enormous potential of AI in decision-making related to child health should not undermine bioethical principles and their application in these priority groups, placing the best interests of children at the center, while respecting at all times the value of the child and the rights to which he or she is entitled.

In the field of AI in pediatrics, there are particular challenges that arise as a consequence of age, race, ethnicity, sex, and level of development. The main issue is the uncritical use of adult data applied to children, resulting in algorithmic bias and the potential for misclassification and inequity because there are marked physiological and anatomical disparities between adults and children and among different pediatric age groups, which necessitate different approaches. Furthermore, children are a research group that is considered particularly vulnerable legally, which requires special ethical consideration.

**Figure 3:**
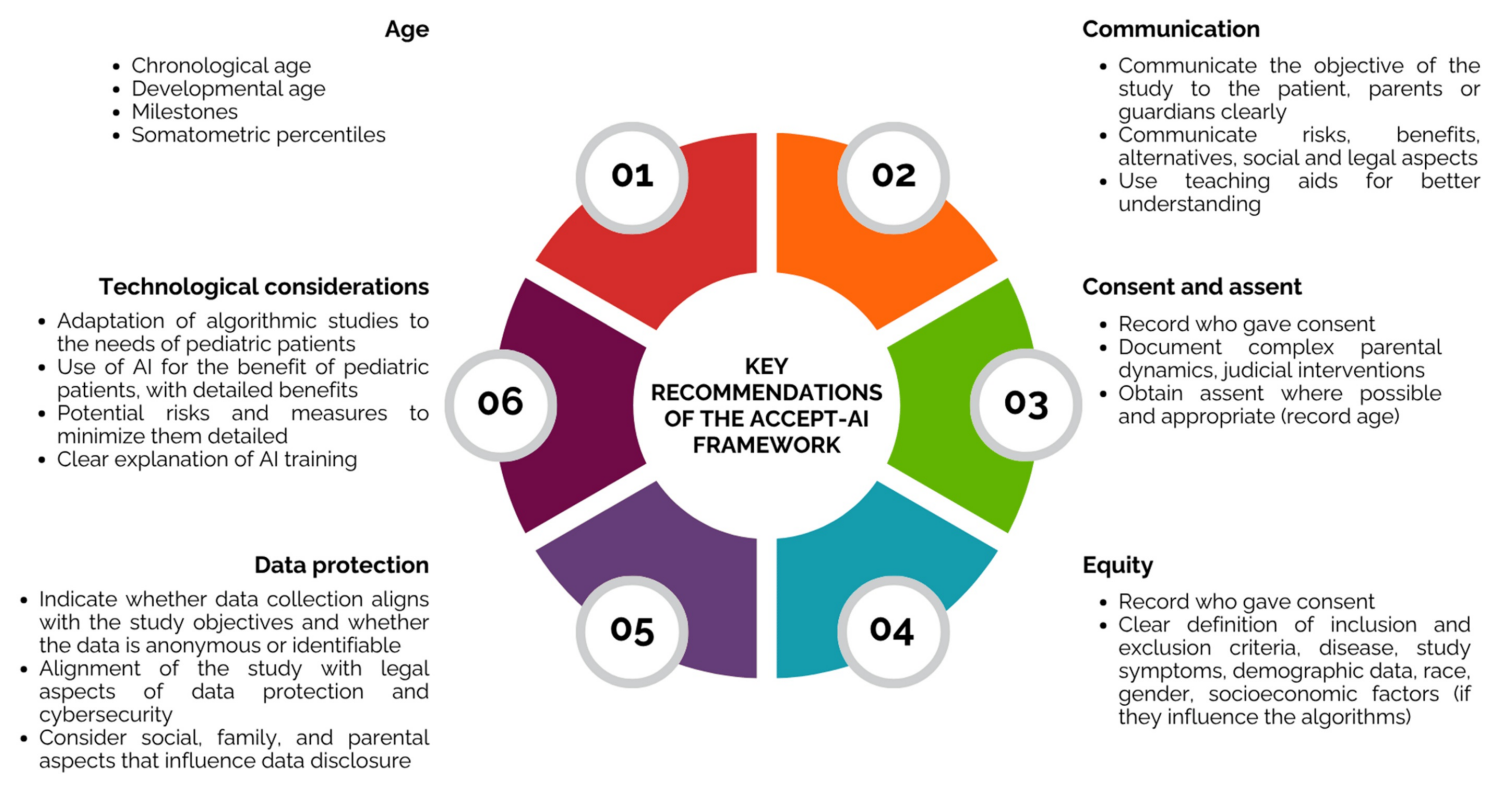
Recommendations of the ACCEPT-AI framework for implementation of AI in pediatric medicine This figure illustrates the six elements of the ACCEPT-AI framework, which represent one possible approach to the ethics of incorporating AI in pediatric healthcare and research. The framework considers age and other relevant special considerations, communication and teaching strategies, informed consent and assent, equitable inclusion, safeguards for children’s data, and transparency of the technology. These factors help ensure that the design, evaluation, and deployment of AI technologies are aligned with the ethical concerns of children and families. The framework also provides measurable recommendations, such as documenting developmental milestones, using communication aids to explain potential risks, obtaining assent, ensuring compliant data protection, and disclosing the pros and cons of the algorithm(s). With this framework, AI applications in pediatric cardiology and related fields can be developed by clinicians, researchers, and developers to be trustworthy and ethically appropriate. Image credits: Created by María-Guadalupe Jiménez-Carbajal using Canva (Canva Pty Ltd., Sydney, Australia). AI: artificial intelligence; ACCEPT: age, communication, consent and assent, equity, protection of data, and technological considerations

In response to these challenges, Stanford University researchers developed age, communication, consent and assent, equity, protection of data, and technological considerations for artificial intelligence (ACCEPT-AI), which articulates a six-part, ethically aligned framework for the use of pediatric data in AI development [16]. Of the six components, the one focused on best practices for reducing bias, improving data diversity, and equitably integrating pediatric population data into algorithmic systems stands out (Figure 3).

More recently, in March 2025, Chng and colleagues, in developing Pediatric Ethical AI Recommendations for Learning and Implementation (AI PEARL), responded to the gap in ethical frameworks pertinent to AI applications in pediatric medicine [17]. Following a systematic evaluation of randomized and non-randomized studies through September 2024, they proposed, consistent with principles of child-centered care, the following ethical guidelines:

The use of AI in pediatric care shall support the child’s development and overall well-being; safety for children in the use of AI in the practice of medicine shall be assured; children and parents/caregivers should be included in the co-design and co-development processes of pediatric AI; equity should be prioritized, and the risks of discrimination should be mitigated; the privacy and confidentiality of children’s data should be protected in the use of AI in the clinical setting; and transparency, explainability, and accountability in the use of AI in pediatrics should be promoted.

These guidelines speak to the immediate need for regulatory, clinical, and technological frameworks that prioritize children, rather than algorithms, as the focal point in advancing pediatric healthcare.

The role of artificial intelligence in pediatric cardiology

**Figure 4:**
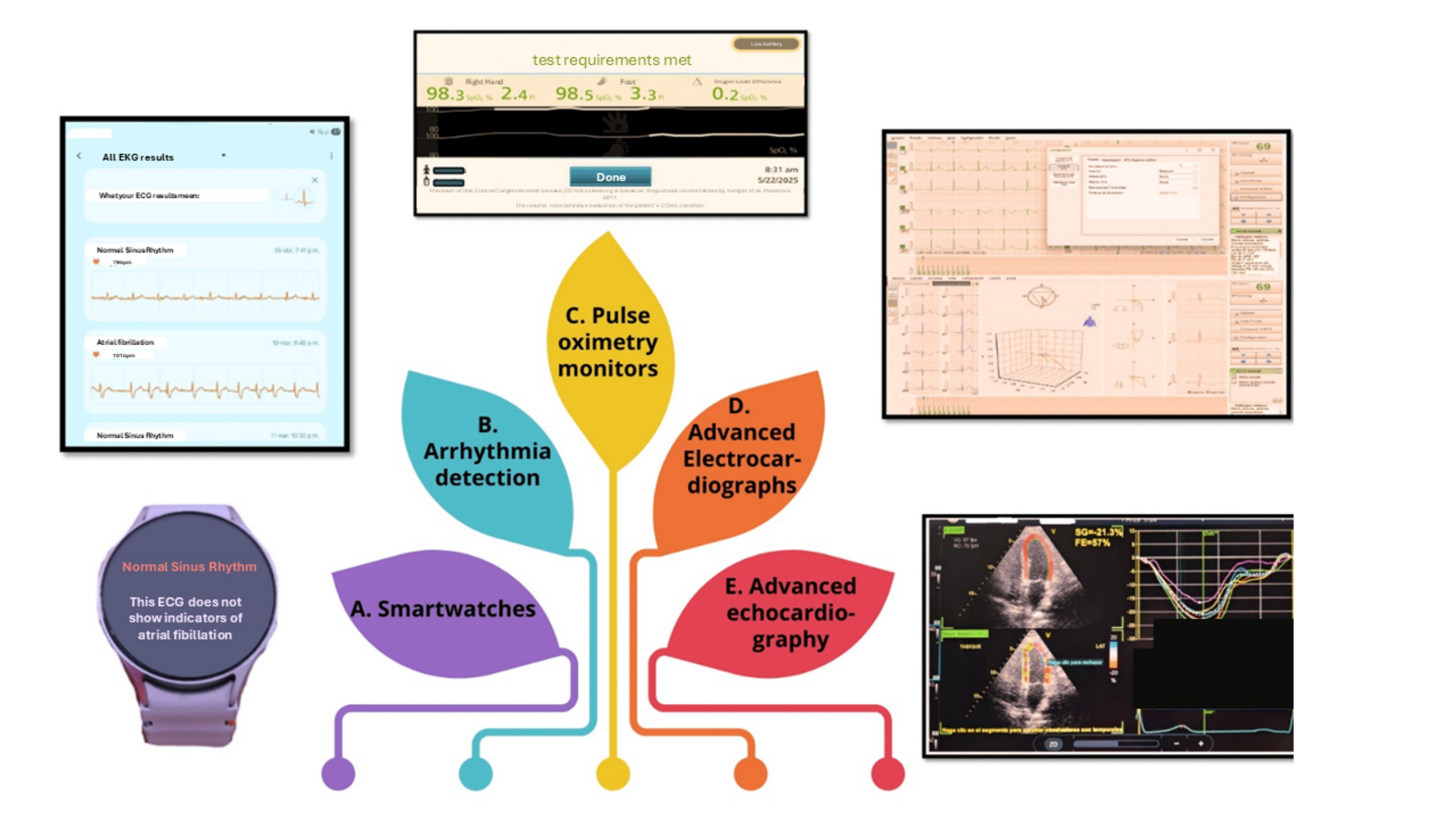
Uses of AI in pediatric cardiology: current technologies for diagnosis, automation, and monitoring This composite figure shows diagnostic and monitoring technologies used in pediatric cardiology that include, or may incorporate, AI-related functions. Smartwatch ECG with automatic rhythm classification (photo of an author's device), mobile ECG (mECG)/smartwatch analysis generating arrhythmia reports (screenshot of a smartwatch report), neonatal screening pulse oximetry using automated, rule-based interpretation of pre-/post-ductal oxygen saturation (screenshot of a pulse oximeter used on an examined neonate), automated ECG analysis with pattern recognition, suggested diagnoses for selected abnormalities (e.g., atrial septal defect), and automated QT/QTc and other measurements (screenshot of a pediatric ECG with advanced QTc analysis), AI-enabled echocardiography with automated quantification of left ventricular GLS to support detection of left ventricular dysfunction in pediatric oncology patients receiving chemotherapy (photo captured during AI-assisted GLS automation by a co-author) are illustrated. These examples demonstrate how AI and automation can support early detection, risk assessment, and longitudinal follow-up in pediatric cardiovascular care. Devices: Samsung Galaxy Watch 7® (Samsung Electronics, Seoul, South Korea), BTL Flexi CardioPoint® (BTL Industries, Hertfordshire, UK), GE Vivid IQ Premium® (GE HealthCare, Chicago, IL, USA), and Masimo Rad-97® (Masimo Corporation, Irvine, CA, USA) (ECG/echocardiogram/pulse oximeter devices owned by the first author and used in her private practice). Source of diagnostic images: original photographs and screenshots taken by the authors from their own devices and anonymized clinical examinations. Image credits: Design by María-Guadalupe Jiménez-Carbajal using Canva (Canva Pty Ltd., Sydney, Australia). AI: artificial intelligence; GLS: global longitudinal strain; ECG: electrocardiography

The use of AI technologies in cardiology subspecialties has also expanded from detecting common arrhythmias with consumer wrist monitors to more sophisticated evaluations of echocardiograms and fetal cardiac ultrasounds. These tools are driven by various supervised and unsupervised learning models to analyze and interpret complex cardiovascular disorders (Figure 4).

Congenital heart defects (CHDs) constitute about 1% of all live births, making them the most common form of congenital defect. Critical congenital heart defects (CCHDs) account for about 25% of all CHDs, and almost all of them are dependent on the ductus arteriosus. These conditions require surgical or interventional correction within the first month of life. They are a major contributor to neonatal morbidity and mortality and represent a public health issue worldwide [18].

In a global systematic analysis conducted between 1990 and 2017, more than 261000 deaths due to CHD were reported. The greatest burden was within low- and middle-income countries [19]. Surgical and diagnostic services have poor availability in those countries, with less than two surgical operating rooms per 100000 population compared to more than 14 in high-income countries [20].

Pediatric cardiology represents a particularly unique specialty in which to consider the use of AI. It has a unique temporal breadth from fetal to adult stages and encompasses the areas of cardiology (echocardiography, MRI, CT, and electrophysiology), interventional cardiology, cardiovascular surgery, intensive care, and cardiac rehabilitation. Furthermore, there is a wide range of cardiac pathologies, and almost every type of congenital anomaly is singular in its presentation. This unique specialty also considers the fact that patients with congenital heart disease have lifelong care needs and represent a high-risk population that requires careful monitoring and sustained follow-up care [21].

Table 1 summarizes the wide range of AI applications within these subspecialties, the clinical relevance of these applications, and the potential to enhance the quality of care, diagnosis, treatment, and administrative efficiencies [18,21].

**Table 1:** Current applications of AI in pediatric cardiology This table outlines the current applications of AI across subspecialties in pediatric cardiology, along with their impact on diagnosis, treatment, and processes. AI: artificial intelligence; ECG: electrocardiography

Domain	AI applications	Clinical impact
Cardiac imaging	AI-enhanced echocardiography, MRI, CT, and 3D reconstruction tools [21,22]	Improved diagnostic precision, reduced radiation dose, faster workflows
ECG	AI algorithms for arrhythmia detection, ischemia screening, and hereditary condition detection [21,22]	Early diagnosis and risk stratification
Planning in interventional cardiology	Use of CT and echocardiographic images superimposed on angiographic images in cardiac catheterizations [21]	Improved diagnostic precision, reduced radiation and contrast medium dose, faster workflows
Surgical planning	AI-based 3D-printed cardiac models and surgical simulation tools [21]	Personalized interventions for complex congenital anomalies
Intensive care monitoring	Predictive AI models for hemodynamic instability and post-operative complication surveillance [21]	Timely interventions and reduced morbidity
Decision support systems	AI-driven risk calculators and treatment recommendation engines [22]	Augmented clinical decision-making and tailored therapy
Resource optimization	AI tools for hospital workflow management and patient prioritization [21]	Increased efficiency and reduced healthcare delivery delays

Sethi and colleagues’ systematic review, published in 2022 and covering literature from 2002 to 2022, noted the surge in the use of AI for creating diagnostic tools in pediatric cardiology, and its applications in the diagnosis of congenital heart disease (CHD), Kawasaki disease, and other acquired heart diseases. The use of AI algorithms in the assessment of electrocardiographs, echocardiography, cardiac CT, and MRI improved the accuracy of diagnosis. The authors noted how predictive algorithms improved surgical and interventional strategies, as well as prognosis [22].

AI-driven software applications also have the capability of creating 3D constructs from the sequence of images obtained and thus may assist in the creation of 3D-printed models of the heart. Such constructs can enhance our comprehension of more intricate congenital heart disease models and can assist in the creation of individualized surgical or interventional plans [21].

Even though electrocardiography (ECG) is one of the oldest methodologies in medicine and medical diagnostics, it has been integrated into AI systems. Algorithms built from extensive datasets have the capability of identifying different types of cardiac rhythm issues, ischemia, and heart attacks, as well as the effects of drugs on the conduction system of the heart.

Deep learning models of pediatric electrocardiograms have been developed to predict cardiac structural and functional alterations, as described by Mayourian et al. in their work published in 2024, in which a convolutional neural network was trained with 92377 pairs of ECG-echocardiograms of patients under 18 years of age with non-severe congenital heart disease, in order to detect dysfunction, hypertrophy, and dilation of the left ventricle (LV) beyond mild, as classified by human experts. The reversal of the lateral T wave, deep S waves in V1 and V2, high R waves in V6, and high R waves in V4 to V6 were recognized as high-risk characteristics. The authors consider the potential of the algorithm for inexpensive screening for left ventricular dysfunction and remodeling in pediatric patients, improving access to care [23].

Emerging technologies are also being considered to expand access to healthcare for pediatric groups within the most vulnerable populations as part of efforts to ensure equity in health services. One of these actions is embodied in the protocol published in October 2025 by Leke and collaborators, which develops a deep learning model for the real-time detection of congenital heart disease in newborns in hospitals in sub-Saharan Africa. The protocol consists of two phases. In the first, the model has been pre-trained since September 2024 with retrospective data from almost 500 newborns. In the second, the model will be adjusted with prospective data from 1000 neonates, with echocardiographic video clips that include 10 standard cardiac views. The work seeks to use a technique that contributes to the early detection of congenital heart disease in environments with limited economic resources [24].

An important challenge is the prenatal detection of congenital heart disease for adequate follow-up planning, birth in specialized centers, adequate postnatal diagnosis, and planned early treatment; thus, the use of AI in fetal cardiology has become important. In the work published by Kim and collaborators, the feasibility of the automated tool HeartAssist was evaluated for the classification of fetal cardiac views, the annotation of cardiac structures, and the measurement of cardiac parameters of fetuses between 20 and 40 weeks of gestation. A total of 65324 images from 2985 fetuses were analyzed. The tool had the ability to classify 10 cardiac views, annotate 26 structures, and measure 43 parameters through convolutional neural networks that were compared with the measurements and annotations of an expert in the field. A classification accuracy of 99.4%, an annotation accuracy of 98.4%, and an automatic measurement success rate of 97.6% were found [25].

Recent technological advances in AI have significantly changed the field of imaging within pediatric cardiology. The most recent form of ultrafast CT reconstruction techniques has the ability to produce high-resolution images while significantly reducing radiation exposure, mitigating motion artifacts, and reducing the need for sedation. Cardiac MRI techniques have been able to shorten the duration of scans and improve image fidelity, which is critical for younger patients who may have anxious episodes during these procedures. In addition, in the last two years, tools have been developed that already consider anatomical and physiological characteristics of specific congenital heart diseases. In this regard, Tilborghs and his team described in 2024 the development and validation of an automatic segmentation and quantification method for the LV and right ventricle (RV) in patients with corrected tetralogy of Fallot, by training a three-dimensional convolutional neural network with cross-validation of short-axis images in manually delineated telediastole and telesystole, obtained from data from patients without cardiac pathology or with acquired pathology, and data from patients with tetralogy of Fallot, finding a marked superiority in right ventricular analysis [26].

The usefulness of integrating imaging techniques with deep learning methods has been observed, as published by Benovoy and his collaborators in 2021, in whose work they sought to establish the relationship between optical coherence tomography findings and vascular compliance through a coronary artery segmentation system based on deep learning in 27 patients with a history of Kawasaki disease. Patients underwent angiography of the left coronary artery during cardiac catheterization, followed by optical coherence tomography of the proximal and distal segments with automatic measurement. The angiographic findings were then compared with those of the tomography, suggestive of vascular damage. On the other hand, 34 patients with coronary aneurysms related to Kawasaki disease, either in regression or persistent over an average time of 14.5 years, were studied with a segmentation framework for the automatic analysis of the temporal evolution of coronary compliance. They contrasted the correlation of the findings of the deep learning method with the severity of the optical coherence tomography findings. The decline in compliance peaked at one year of evolution [27].

Dimensions of quality of care and ethical aspects

The definition of quality in healthcare has shifted through multiple frameworks and disciplines. One of the most encompassing is the definition from Dr. Héctor Aguirre-Gas, describing quality of care as the provision of health services that are timely, competent, safe, ethically sound, and that meet the clinical and emotional expectations of the patient [28].

Avedis Donabedian is known as the father of quality of care and the first to develop a systematic approach to evaluating the quality of care in 1961. This approach has been adapted and adopted in nearly every healthcare system in the world. He identified three components of every healthcare system that are interrelated: structure (both physical and organizational resources), process (both clinical and service delivery), and outcome (health states of the patient as a result) [29].

The Institute of Medicine (IOM) also built on this model. In the 2001 report Crossing the Quality Chasm, the IOM outlined six of the fundamental goals of quality improvement in health care: effectiveness, whereby services are provided based on scientific evidence and delivered by qualified professionals; safety, where care is delivered without the occurrence of preventable errors; timeliness, in which delays are minimized for health service users as well as for service providers; equity, to guarantee equal access to care regardless of the patient’s sex, race, socio-economic status, or geographical region; patient-centeredness, which expresses the importance of recognizing the individual needs and preferences of the patient; and efficiency, which looks at the optimal use of resources to eliminate time, material, and effort waste [30].

These objectives can be evaluated against the four principles of bioethics: autonomy, beneficence, non-maleficence, and justice. Together with the quality indicators, these objectives can be used to develop a framework to assess pediatric cardiology (especially with the use of AI).

In 2025, the WHO published a document that provides guidance on the measurement and monitoring of the quality of care, with a focus on improving maternal, newborn, child, and adolescent health services, and which proposes indicators for evaluating the quality of healthcare and access throughout these crucial stages of life [31]. These indicators not only provide the WHO with vital information to assess the effectiveness of a country's health system but also support the adoption and ensure the fair and equitable use of AI in a clinical and public health context.

A primary contemporary focus in quality measurement is the use of electronic health records (EHRs) integrated with data systems, many of which are driven by AI and other forms of machine learning to streamline and automate the analysis of clinical workflows and outcomes. This type of integration is relevant to quality performance assessment, auditing, and the iterative improvement cycle.

Artificial intelligence agents and ethical risk in quality-driven systems

The International Business Machines Report on Artificial Intelligence Agents

Opportunities, Risks, and Mitigation describes AI agents as software entities that can make decisions autonomously to achieve specified goals [30]. AI agents are able to enhance productivity and efficiency in a variety of healthcare functions, including diagnosis, scheduling, and allocation of resources.

The report mentions some of the most notable ethical and operational risks and challenges that could arise from the implementation of agents, among which are misalignment of values, a situation in which they can act against human morality, ethics, guidelines, or policies; impartiality, which implies discriminatory biases derived from the learning of biased information; excessive or misdirected trust on the part of users; computational inefficiency that occurs due to redundant and even unnecessary actions that the AI agent can execute to reach a goal, which could be insufficient or even harmful; security vulnerabilities, where systems may be exposed to hacking, system modifications, or data loss; violation of privacy and intellectual property through the distribution of personal and confidential information to users or to other tools or agents; and lack of explainability, a situation in which AI systems can make arbitrary or misleading decisions that are not easily traceable. The result could be a "black box" phenomenon.

The need for ethically aligned systems to be built and tested, and then closely monitored, is highlighted by the above concerns. The right strategies must be in place to address challenges of transparency, legal accountability, auditing, and interoperability of systems.

Positive and negative impacts of artificial intelligence on pediatric cardiology care

Quality and Efficiency Potential Gains

AI in pediatric cardiology has the potential to increase the quality and efficiency of care in high-complexity settings, with a high level of specialized expertise needed. AI has the potential to transform the diagnostic landscape by spotting clinically significant characteristics in data that surrounding experts may find hard to see. Supervised and unsupervised learning-based systems enhance treatment plans by enabling earlier and more precise diagnoses and thus better clinical results.

AI assists in predictive analytics and the optimization of data resources and real-time analysis. Staff, equipment, and facility resources can all be optimized to a greater degree. These advancements may be able to reduce the amount of manual work that healthcare professionals do, allowing them to spend more time with patients.

An AI system in pediatric cardiology must adhere to ethical principles, user training, and system transparency. Without these, there is a greater chance that the quality of care will worsen rather than improve.

Ethical Barriers and Perception of Vulnerability

The incorporation of AI in pediatric cardiology is promising, but the most significant barriers continue to be ethical and socio-cultural.

Children have an elevated level of vulnerability, not only because of their age and stage of development, but because guardians, who usually have to make difficult clinical decisions, are put in positions of uncertainty or stress as a result of the complexity and range of technological options available.

These and other ethical concerns (such as the potential for algorithmic discrimination, data security, the capacity for truly informed consent, and equity of access to care) require a shift in focus. These are the challenges of the responsible use of these technologies in clinical settings.

Table 2 summarizes such challenges and offers a range of strategies to promote trust, safety, and ethics in their use to integrate AI technologies in ways that are respectful and responsive to the needs of patients.

**Table 2:** Ethical challenges of AI in pediatrics: suggested strategies Ethical challenges related to the use of AI in pediatric cardiology and strategies to address them, with a focus on the child. Recommendations of the ACCEPT-AI framework for implementation of AI in pediatric medicine. AI: artificial intelligence; CHD: congenital heart defect; ACCEPT: age, communication, consent and assent, equity, protection of data, and technological considerations

Ethical challenge	Example in pediatric cardiology	Proposed mitigation strategy
Algorithmic bias	Underrepresentation of complex CHDs in AI training	Incorporate diverse and representative pediatric datasets [16]
Transparency (“black box” problem)	AI-based diagnostic decisions without clinician interpretability	Develop explainable AI models and involve physicians in decision loops [16,17]
Informed consent and assent	Difficulty obtaining consent for AI use in infants and young children	Develop child-appropriate communication tools and family-centered consent [16,17]
Data privacy and security	Risk of breaches in sensitive pediatric health records	Implement advanced encryption and strict governance frameworks [16,17]
Health equity gaps	Limited AI access in low-resource pediatric cardiac care settings or difficult access to health services	Promote global policy frameworks for equitable AI distribution [16,17]
Medical education	Limited knowledge of AI tools among healthcare personnel who care for children with heart disease	Optimization of doctor-patient-family communication, health literacy, and knowledge generation through research on the use of AI in pediatric cardiology [16,17]

In the cross-sectional study led by Berghea and published in 2023, caregivers' perceptions regarding the use of AI in outpatient pediatric care within ambulatory, emergency, surgical, and imaging pediatric care areas were analyzed, as well as their perceptions regarding diagnostic processes in the pediatric emergency room in three monosectional tertiary pediatric hospitals in Romania. Acceptance of AI showed different levels of trust depending on the educational level of the caregivers. Among caregivers, 22.2% of those with a graduate degree declared their rejection, compared with 43.9% of those with an undergraduate degree and 54.5% of those with a high school diploma. Additionally, 70.1% stated that the potential for medical errors caused by AI was a major concern. The majority of respondents had a negative view of AI, but under the supervision of a doctor, the use of AI was supported as long as it was explained to families. The use of AI was proposed on the basis of the ethics of consent to carry out procedures [32].

It was unexpected that only 17.5% of caregivers stated concern that AI would pose a risk to productivity and act as an obstacle to accessing care; 34.1% of respondents stated that they were concerned about data privacy. Caregivers prioritized having the option to accept or decline AI decisions over the right to equitable access to such technologies. This suggests that the implementation of AI technologies in vulnerable pediatric populations requires additional ethical training and policies.

Structural Inequities and the Role of Consent

Previous lines have highlighted the advances and beneficial prospects of the use of AI in pediatric cardiology; however, despite technological advances, the distribution of sophisticated diagnostic tools and treatment options remains uneven, as it is documented that around 4.8 billion people still do not have access to affordable and safe surgical services, most of whom live in resource-poor settings [33].

Pediatric cardiology is a prime example of this. Specialized procedures are often only performed at tertiary centers, which separates families, creates financial burdens, lengthens hospital stays, and adds additional logistical complexities. This is especially true in health systems that are centralized and have a limited distribution of pediatric cardiology services, as is the case in some parts of Mexico and Latin America.

Ethical frameworks such as ACCEPT-AI help to promote the just inclusion of pediatric populations in the development of algorithms, including pediatric data. Still, many institutions lack formal protocols for seeking assent from older children or adolescents, despite their cognitive capacity to participate in decisions affecting their health.

This illustrates a lack of justice in digital health. The inequitable distribution of access, data security, and communication in digital health remains unaddressed. Providers have to strengthen the practice of communication, the exercise of shared decision-making, and the inclusion of consent mechanisms that encompass both the exercise of parental authority and the autonomy of the child.

AI must be accurate, efficient, and ethically responsible. It is the developer's responsibility to ensure that interventions are aligned with the 7Ps (Predictive, Preventive, Personalized, Accurate, Participatory, and Polyprofessional) and PEARL-AI frameworks, which in this case prioritize equity, child agency, and transparency [15,17].

## Conclusions

AI will not replace clinical judgment in pediatric cardiology, although clinical judgment will be necessary when using AI to aid in diagnosis, risk assessment, and follow-up. Clinicians will be responsible for making clinical decisions and also for how AI results are used in patient care. The problem of age bias persists; pediatric data remain underrepresented, heterogeneous, and do not include children with more complex or atypical CHDs. This, unfortunately, leads to less effective models in actual pediatric use. To improve the situation, the focus should be on the representativeness of datasets, guidance for clinicians on the appropriate and responsible use of AI to avoid over-reliance on results, as well as on effective communication and shared decision-making with families in states of uncertainty. In Mexico and other low- and middle-income settings, the unequal distribution of pediatric cardiovascular services poses additional obstacles to implementation. However, ethically focused AI is designed to help bridge gaps, improving and strengthening triage pathways, image analysis, and specialist input into routine workflows.

To achieve these benefits, policies and institutions must align and prioritize the rights of children and adolescents, and the objectives of quality, safety, timeliness, equity, transparency, auditability, and patient-centered care. According to the present narrative review, reality underscores the need to rapidly scale up equitable, high-level, AI-integrated care for children with cardiovascular disease. Although technological tools offer significant potential, they must be accompanied by policies that address bioethical considerations, staff shortages, unequal distribution of services, and financial barriers.
